# Comprehensive analysis of copy number variance and sensitivity to common targeted therapy in clear cell renal cell carcinoma: In silico analysis with in vitro validation

**DOI:** 10.1002/cam4.3281

**Published:** 2020-07-06

**Authors:** Yuqing Li, Yanyun Shen, Zhidong Zhu, Hui Wen, Chenchen Feng

**Affiliations:** ^1^ Department of Urology Huashan Hospital Fudan University Shanghai PR China; ^2^ Department of Dermatology Huashan Hospital Fudan University Shanghai PR China; ^3^ Department of Cardiology Huashan Hospital Fudan University Shanghai PR China

**Keywords:** clear cell renal cell carcinoma, copy number variance, resistance, tyrosine kinase inhibitor

## Abstract

**Background:**

Chromosomal rearrangements are common in clear cell renal cell carcinoma (ccRCC) and their roles in mediating sensitivity to tyrosine kinase inhibitors (TKIs) and mTOR inhibitors (mTORi) remain elusive.

**Methods:**

We developed an in silico strategy by screening copy number variance (CNV) that was potentially related to TKI or mTORi sensitivity in ccRCC by reproducing the TCGA and GDSC datasets. Candidate genes should be both significantly prognostic and related to drug sensitivity or resistance, and were then validated in vitro.

**Results:**

ADCYAP1 loss and GNAS gain were associated with sensitivity and resistance and to Cabozantinib, respectively. ACRBP gain and CTBP1 loss were associated with sensitivity and resistance and to Pazopanib, respectively. CDKN2A loss and SULT1A3 gain were associated with sensitivity and resistance and to Temsirolimus, respectively. CCNE1 gain was associated with resistance to Axitinib and LRP10 loss was associated with resistance to Sunitinib. Mutivariate analysis showed ADCYAP1, GNAS, and CCNE1 remained independently prognostic when adjusted for the rest.

**Conclusion:**

Here we show CNVs of several genes that are associated with sensitivity and resistance to commonly used TKIs and mTORi in ccRCC. Further validation and functional analyses are therefore needed.

## Graphical abstract

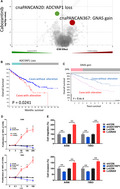

## 1 BACKGROUND

The clear cell renal cell carcinoma (ccRCC) is in histopathology statistically accounting for more than 70% of renal cell carcinoma, which takes blame for diagnosis and deaths of 2% from all the carcinomas worldwide.^1^ The tumorigenesis emerges firstly from renal tubular epithelial cell and grow as the similar morphology as that. Compared with the subtypes of collecting duct carcinoma and undifferentiated carcinoma, ccRCC shows an average malignancy. It has come to a consensus that the inactivation of VHL gene, a suppressor gene relevant with cellular oxygen sensing, does business for the originality of tumor growth. VHL alteration could be found in various diseases not only retinal hemangioblastomas, pancreatic neuroendocrine tumors but also ccRCCs.^2^ The ccRCC is also believed as a metabolic disease for the revision of glucose and fat metabolism, bringing us new dimensions to know about the prime and development of the cancer and dig out more targets for the disease.^3^

Copy number variations (CNVs) are of great significance to the tumorigenesis, together with the gene mutation, transcription changes, translation adjusts, and epigenetic modification. Compared with other genetic diversification, CNV is more likely to lead to irregular gene structure, which includes gene loss, deletion, amplification, and gain, resulting in the activation of oncogenes or inactivation of suppressors.^4^ Hieronymus mentioned that to what degree the CNVs affect the genome depends on the changed fragment proportion of the whole length of DNA, which is called tumor CNV burden academically and chronically. Actually, it has been identified that tumor CNV burden was deeply correlated with recurrence and prognosis of tumors, especially the prostate cancer.^5^ Moreover, via the disturb in signaling pathways, CNV was also found significant imply for the diagnosis and prognosis in patients with gastric cancers.^6^

Based on a research on a group of patients with ccRCC, it was firmly identified that CNV, whether displayed as loss or gain of genome, occurs in thousands of genes involved in diverse signaling pathways like KEGG and could be applied in the stratification of Fuhrman grade and guidance value for prognosis and prediction.^7^ Another larger scale study was also engaged in CNV analysis so as to recognize the targeted gene and came to a summary that loss of 3p, gain of 5q, and gain of 7q accounts for the most of lesions.^8^ Recently, MDPZ was classified coming up CNV with about 30% in ccRCC patients and the deletion rate increases along with the aggravation of cancer. Furthermore, the loss of MDPZ predicts a worse prognosis with a shorter survival performance in Kaplan‐Meier curves.^9^

In the current study, we have investigated associations between sensitivity to commonly used tyrosine kinase inhibitors (TKIs) and CNVs in ccRCC using an in silico exploration with in vitro validation.

## 2 MATERIALS AND METHODS

### 2.1 In silico analysis

#### 2.1.1 Reproduction of Genomics of Drug Sensitivity in Cancer (GDSC) dataset

The GDSC dataset was used to select candidate focal deletion or amplifications that were associated with sensitivity to TKIs including cabozantinib, pazopanib, temsirolimus, axitinib, and sunitinib. Considering statistical significance did not necessarily translated to biological effect, we only designate significance using the *P* value and not the *q* value (false discover rate). By entering the GDSC interface, we queried sensitivity data of the TKI and among all genetic data we only focused on CNVs labeled as cnaPANCAN. CNVs that were associated with sensitivity or resistance (*P* threshold of < 0.05) were then investigated for association with survival. Volcano plots were automatically generated by GDSC.

#### 2.1.2 Reproduction of The Cancer Genome Atlas (TCGA) dataset

TCGA‐KIRC dataset was analyzed and reproduced with the cBioPortal platform. Candidate genes from GDSC were submitted to the cBioPortal website interface for CNV status. Type of alteration (CN loss or gain) corresponded to that of cnaPANCAN in GDSC. In TCGA, we queried both heterozygous loss (HETLOSS) and homozygous deletion (HOMDEL) of the gene that were defined as CN loss in GDSC, and both amplification (AMP) and gain (GAIN) of the gene that were defined as CN gain in GDSC. Survival profiles including Kaplan‐Meier curve and log‐rank test were generated by cBioPortal. For focal alteration that encompassed several genes as shown in cnaPANCAN, we queried only genes showing corresponding mRNA change in the “Plot” function of cBioPortal. To further validate the findings, we also queried mRNA expression level of the gene against survival in the Human Protein Atlas platform.

### 2.2 In vitro assays

Candidate genes that were associated both with TKI sensitivity and survival were tested for in vitro sensitivity to the corresponding compounds. As we confirmed CNV of the candidate genes corresponded to mRNA expression in TCGA dataset, we used RNA interference to mimic CNV, namely knock‐down (KD) for CN loss and overexpression (OE) for CN gain.

#### 2.2.1 Cell lines

Both 786O and A498 ccRCC cells were purchased from the cell bank of Chinese Academy of Science and cultured in RPMI‐1640 medium. Both cells were established model cells for ccRCC and were extensively used worldwide. The cDNA clones for lentiviral delivery of GNAS, ACRBP, SULT1A3, and CCNE1 were obtained from Origene. We used TRC (TRC, http://www.broadinstitute.org/rnai/public/) to construct shRNA targeting ADCYAP1 (TRCN0000371228), CTBP1 (TRCN0000273905), CDKN2A (TRCN0000255853), and LRP10 (TRCN0000063424). Infection protocol of lentivirus was well established.^10^ Vectors with resistance to puromycin were constructed and transfected via nonlipofectamine Fugene transfection. After incubation, positive clones were selected by puromycin supplement and control vectors were generated with similar approach.

#### 2.2.2 Quantitative polymerase chain reaction (qPCR)

All interfered genes were tested for mRNA expression level using quantitative PCR (qPCR) Total RNA was extracted with Trizol reagent and was converted to cDNA. Primers were designed as per PrimerBank (https://pga.mgh.harvard.edu/cgi‐bin/primerbank) as follows: ADCYAP1 (PrimerBank ID 153266791c1), GNAS (PrimerBank ID 4504047a2), ACRBP (PrimerBank ID 17999523c1), CTBP1 (PrimerBank ID 61743966c1), CCNE1 (PrimerBank ID 339275820c1). SULT1A3 was not included in PrimerBank and the primers were designed as follows: forward primer, 5′‐GGA ACC CTC AGG GCT GGA G‐3′ and reverse primer, 5′‐CGT CCT TTG GGT TTC GGG‐3′. Reactions were run on an ABI7500 device and all samples were run in triplicates.

#### 2.2.3 Proliferation assay

Cell proliferation was measured using the crystal violet assay. Gradient concentration of each compound used in treatment was referred to reported IC50 values in GDSC dataset. For lacking data, literature‐reported IC50 values were used. All measurements were performed at 96 h of treatment. Cells were seeded at the density of 2500 cells/well and were subsequently treated with crystal violet. Ten percent of formalin were used for cell fixation after removal of medium. Methanol was used and plates were read at absorbance at 540 nm. Proliferation was presented as percentage in relation to control arm.

#### 2.2.4 Transwell assays

Both invasion and migration were measured by Transwell assay. Cells were seeded in the upper chamber of the Transwell plate at the density of 1 × 10^6^/ml, either coated (for invasion) or uncoated (for migration) with Matrigel. Upper chamber was supplemented with serum‐free media while the lower chamber was filled with complete medium. Cells that penetrated were stained with crystal violet and counted for number.

#### 2.2.5 Statistical analysis

Volcano plots were generated automatically by the GDSC platform. Survival was plotted using the Kaplan‐Meier curves and analyzed using the log‐rank test. The two‐way ANOVA was used to compare proliferation between groups. Multivariate analysis was performed using the Cox regression model. The value of *P* < .05 was accepted as statistically significant.

## 3 RESULTS

### 3.1 Sensitivity and resistance to Cabozantinib

**FIGURE 1:**
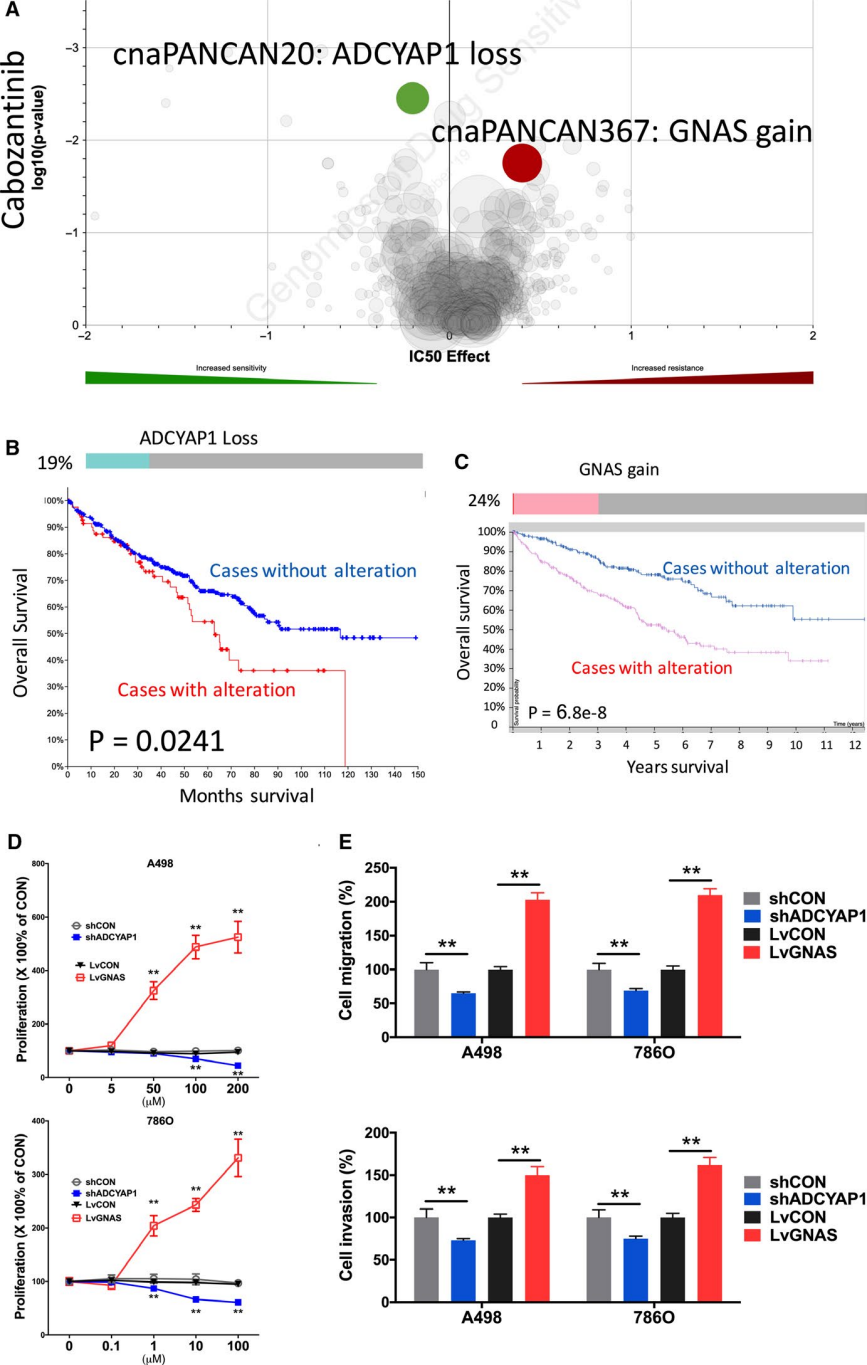
Sensitivity and resistance to Cabozantinib. Shown were (A) volcano plot reproduced from GDSC dataset showing sensitivity and resistance to Cabozantinib; (B) Oncoprint of CNV of ADCYAP1 reproduced from TCGA dataset and analyzed using cBioPortal together with overall survival curves for CNV altered cases; (C) Oncoprint of CNV of GNAS reproduced from TCGA dataset and analyzed using cBioPortal together with survival curves for mRNA expression with automatically designated cutoff value; (D) proliferation assay and (E) Transwell migration and invasion assays showing response to different doses of Cabozantinib in two ccRCC cell line (*P < .05; **P < .01)

Cabozantinib stood at second‐line treatment of mccRCC recommended by the EAU. We found that CN loss of ADCYAP1 was associated with sensitivity to Cabozantinib (Figure 1A). Heterozygous loss of ADCYAP1 occurred in 19% of ccRCC cases and was significantly associated with worsened overall survival (Figure 1B). Gain of GNAS was associated with resistance to Cabozantinib (Figure 1A). Gain of GNAS was present in 24% of cases with one amplification case (Figure 1C). Gain of GNAS was significantly associated with worsened overall survival (Figure 1C). ADCYAP1‐KD showed significant inhibited proliferation and GNAS‐OE showed significant increased proliferation in both ccRCC cells (Figure 1D). Similar trend was also observed in invasion and migration assays **(**Figure 1E).

### 3.2 Sensitivity and resistance to Pazopanib

**FIGURE 2:**
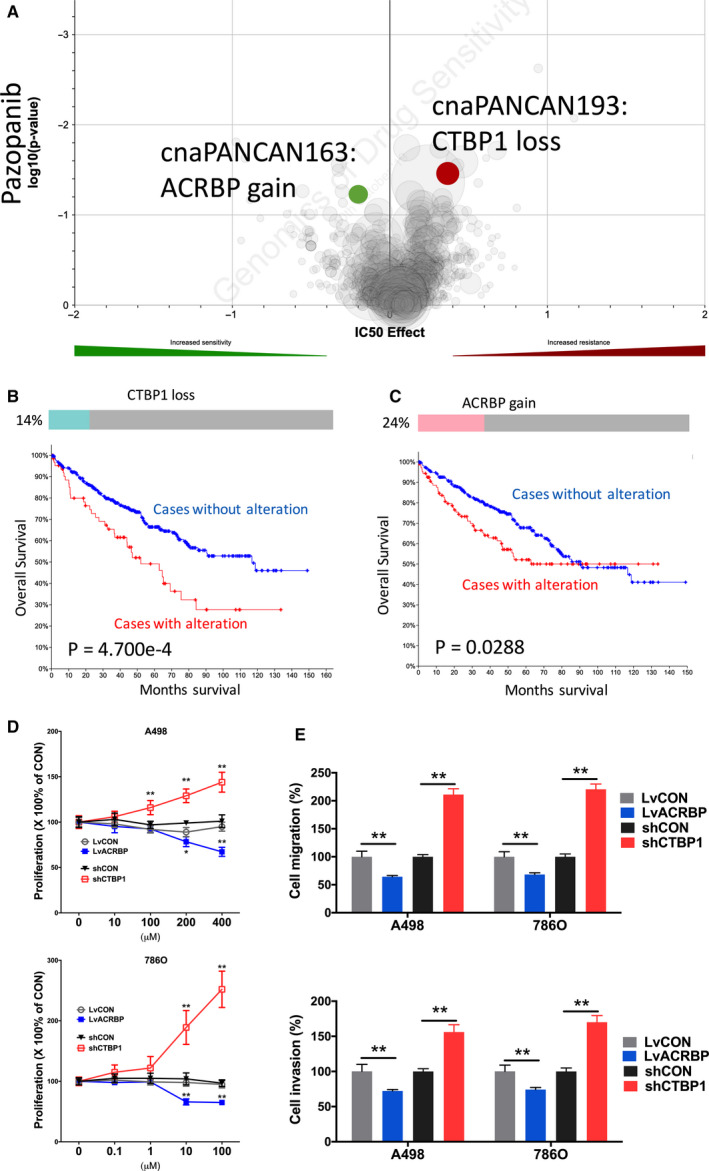
Sensitivity and resistance to Pazopanib. Shown were (A) volcano plot reproduced from GDSC dataset showing sensitivity and resistance to Pazopanib; (B) Oncoprint of CNV of ACRBP reproduced from TCGA dataset and analyzed using cBioPortal together with overall survival curves for CNV altered cases; (C) Oncoprint of CNV of CTBP1 reproduced from TCGA dataset and analyzed using cBioPortal together with survival curves for CNV altered cases; (D) proliferation assay and (E) Transwell migration and invasion assays showing response to different doses of Pazopanib in 2 ccRCC cell lines. (*P < .05; **P < .01)

Pazopanib stood at first‐line treatment of mccRCC recommended by the EAU. We found that CN gain of ACRBP was associated with sensitivity to Pazopanib (Figure 2A). Heterozygous loss of CTBP1 occurred in 14% of ccRCC cases and was significantly associated with worsened overall survival (Figure 2B). Gain of ACRBP was associated with resistance to Pazopanib (Figure 2A). Gain of ACRBP was present in 24% of cases with one amplification case (Figure 2B). Higher expression of ACRBP was associated with worsened prognosis (Figure 2C). CTBP1‐KD showed significant inhibited proliferation and ACRBP‐OE showed significant increased proliferation in both ccRCC cells (Figure 2D). Similar trend was also observed in invasion and migration assays (Figure 2E).

### 3.3 Sensitivity and resistance to Temsirolimus

**FIGURE 3:**
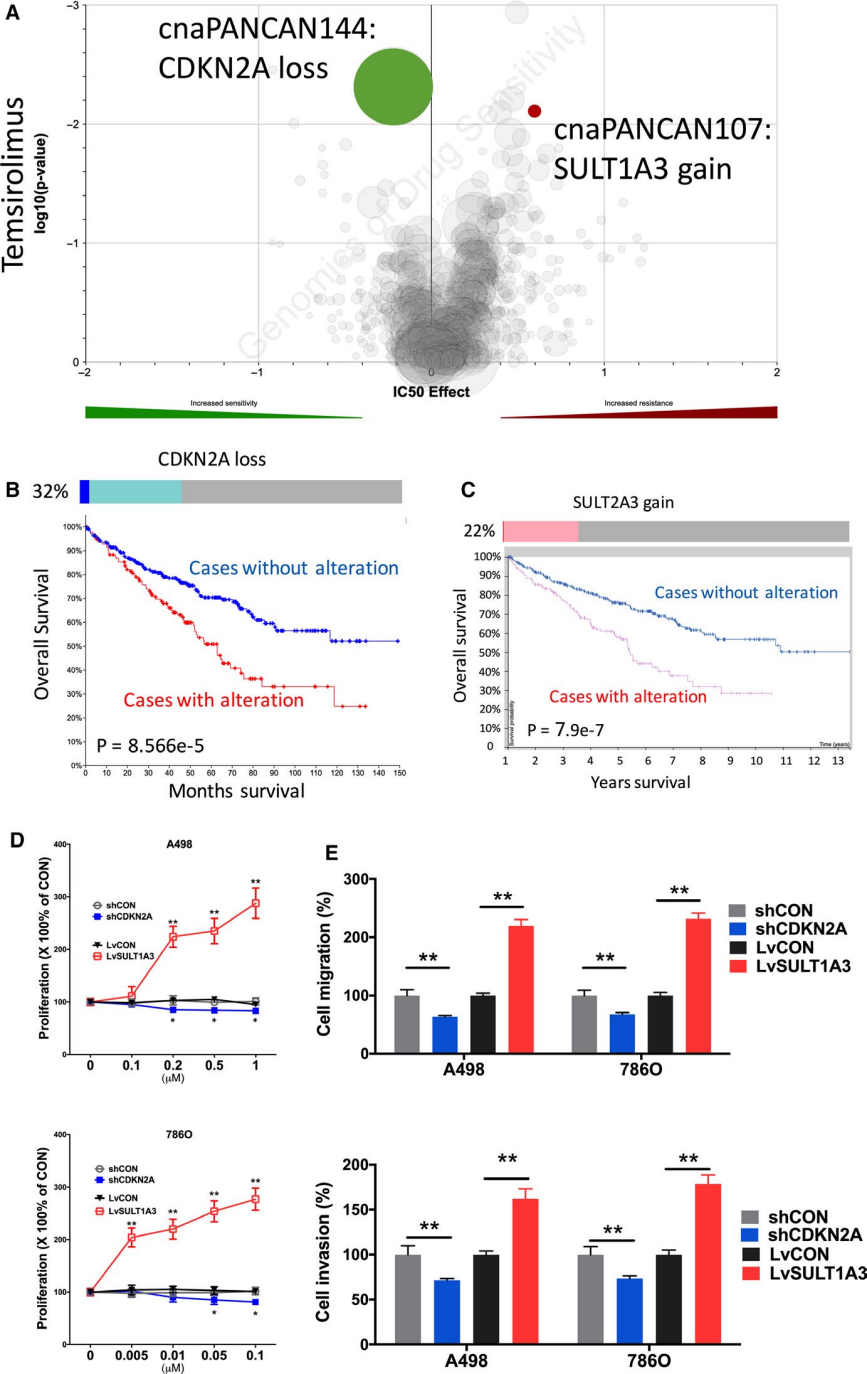
Sensitivity and resistance to Temsirolimus. Shown were (A) volcano plot reproduced from GDSC dataset showing sensitivity and resistance to temsirolimus; (B) Oncoprint of CNV of CDKN2A reproduced from TCGA dataset and analyzed using cBioPortal together with survival curves for CNV altered cases; (C) Oncoprint of CNV of SULT1A3 reproduced from TCGA dataset and analyzed using cBioPortal together with survival curves for mRNA expression with automatically designated cutoff value; (D) proliferation assay; and (E) Transwell migration and invasion assays showing response to different doses of Temsirolimus in two ccRCC cell lines. (*P < .05; **P < .01)

Temsirolimus stood at third or later lines of treatment of mccRCC recommended by the EAU. Gain of SULT1A3 was associated with resistance to Temsirolimus (Figure 3A). Gain of SULT1A3 was present in 22% of cases with one amplification case (Figure 3B). Higher expression of SULT1A3 was associated with worsened prognosis (Figure 3B). We found that CN loss of CDKN2A was associated with sensitivity to the Temsirolimus (Figure 3A). Heterozygous or homozygous loss of CDKN2A occurred in 32% of ccRCC cases and was significantly associated with worsened overall survival (Figure 3C). CDKN2A‐KD showed significant inhibited proliferation and SULT1A3‐OE showed significant increased proliferation in both ccRCC cells (Figure 3D). Similar trend was also observed in invasion and migration assays (Figure 3E).

### 3.4 Resistance to Axitinib

**FIGURE 4:**
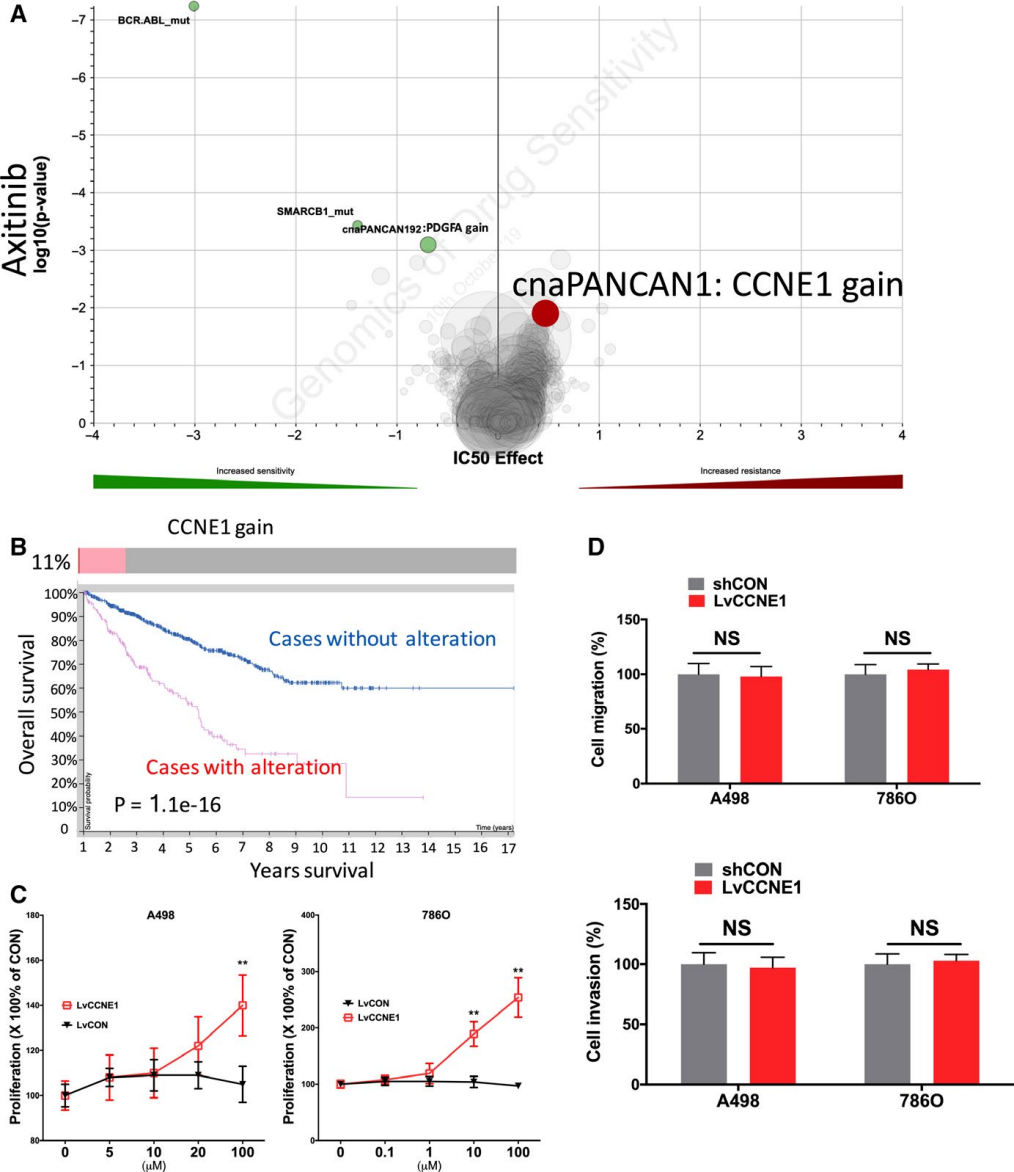
Sensitivity and resistance to Axitinib. Shown were (A) volcano plot reproduced from GDSC dataset showing sensitivity and resistance to Axitinib; (B) Oncoprint of CNV of CCNE1 reproduced from TCGA dataset and analyzed using cBioPortal together with survival curves for CNV altered cases; (C) proliferation assay and (D) Transwell migration and invasion assays showing response to different doses of Axitinib in two ccRCC cell lines. (*P < .05; **P < .01)

Axitinib stood at second‐line treatment of mccRCC recommended by the EAU. Gain of CCNE1 was associated with resistance to Axitinib (Figure 4A). Gain of CCNE1 was present in 11% of cases with one amplification case (Figure 4B). Higher expression of CCNE1 was associated with worsened prognosis (Figure 4B). CCNE1‐OE showed significant increased proliferation in both ccRCC cells (Figure 4C). However, CCNE1 status did not change ability of migration or invasion of ccRCC cells **(**Figure 4D).

### 3.5 Resistance to Sunitinib

**FIGURE 5:**
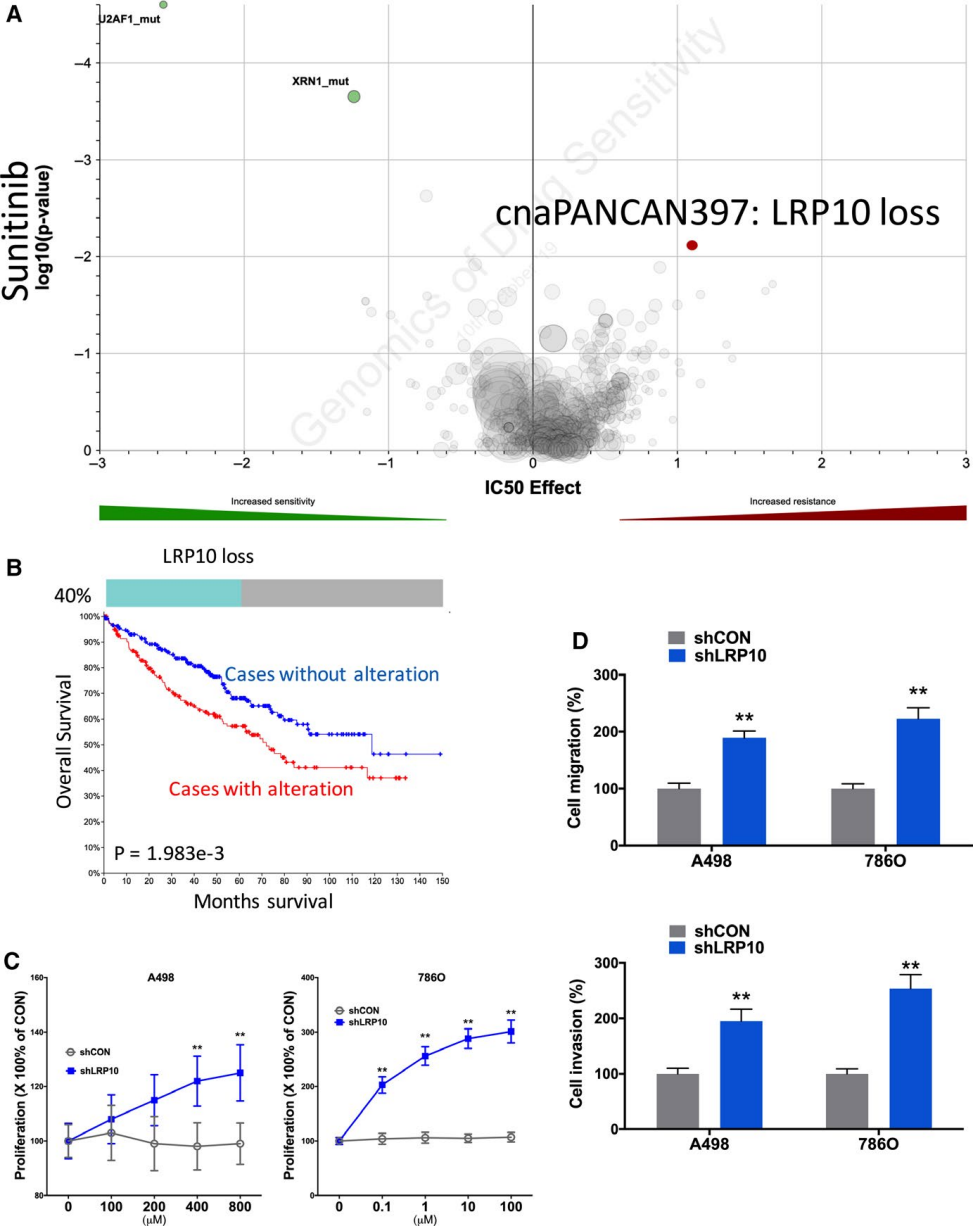
Sensitivity and resistance to Sunitinib. Shown were (A) volcano plot reproduced from GDSC dataset showing sensitivity and resistance to Sunitinib; (B) Oncoprint of CNV of LRP10 reproduced from TCGA dataset and analyzed using cBioPortal together with survival curves for CNV altered cases; (C) proliferation assay and (D) Transwell migration and invasion assays showing response to different doses of sunitinib in two ccRCC cell lines. (*P < .05; **P < .01)

Sunitinib stood at first‐line treatment of mccRCC recommended by the EAU. We found that CN loss of LRP10 was associated with resistance to Sunitinib (Figure 5A). Heterozygous loss of LRP10 occurred in 40% of ccRCC cases and was significantly associated with worsened overall survival (Figure 5B). LRP10‐KD showed significant inhibited proliferation in both ccRCC cells (Figure 5C). Similar trend was also observed in invasion and migration assays **(**Figure 1E).

As all genes were validated to be prognostic, we then performed multivariate analysis to adjust the prognostic contribution of each gene. We found that ADCYAP1, GNAS. and CCNE1 were independent prognostic while the rest turned statistically insignificant (Table 1).

**TABLE 1:** Multivariate analysis (Cox regression) selected genes in the current study and their contribution to survival, reproduced from the TCGA‐KIRC (renal clear cell carcinoma) dataset

	Coefficient	HR	95% Confidence interval		*P* value
			Lower	Upper	
ADCYAP1	–0.438	0.646	0.48	0.868	0.004
GNAS	0.39	1.478	1.11	1.967	0.008
ACRBP	0.027	1.027	0.808	1.306	0.825
CTBP1	0.098	1.103	0.669	1.819	0.7
CDKN2A	0.052	1.054	0.901	1.233	0.512
SULT1A3	0.19	1.209	0.959	1.524	0.109
CCNE1	0.437	1.547	1.252	1.912	<0.0001
LRP10	–0.19	0.827	0.642	1.066	0.142

## 4 DISCUSSION

In the current study, we have comprehensively analyzed CNV in ccRCC and the associations with sensitivity to TKIs and mTORi used clinically. Surprisingly, most of CNVs in our findings were not mechanistically related to effecting pathway of the corresponding TKI.

**FIGURE 6:**
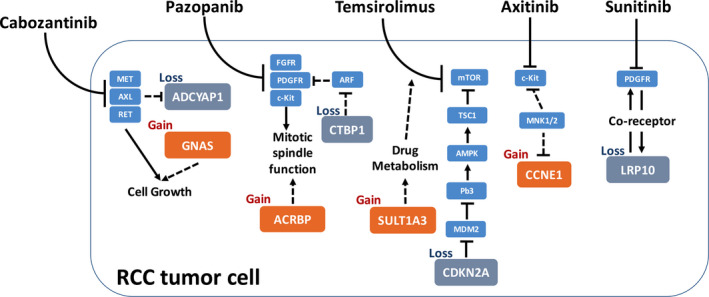
Schematic chart of drug sensitivity‐related genes in the current study with proposed interaction or regulatory pathway in ccRCC. Solid lines represent reported interaction. Dashed lines represent proposed interaction. Arrows indicate stimulatory and T‐shapes indicate inhibitory

We showed sensitivity to Cabozantinib was associated with ADCYAP1 loss and GNAS gain. ADCYAP1 gene encodes adenylate cyclase activating polypeptide 1 that improves the cAMP levels, activating the transcription of target gene. ADCYAP1 was detected secreted from hypothalamic neurons and functions in pituitary cell membranes as a protein kinase A signaling pathway activator.^11^ Shintani also put forward the significance of ADCYAP1 in psychomotor function and described the inhibition in jumping behavior seen in mice lacking PACAP (Adcyap1‐/‐).^12^ It has been shown on GeneCards that Cabozantinib target AXL inhibits ADCYAP1 with an association score of 0.86, which in part explains the sensitivity of ADCYAP1 loss of Cabozantinib (Figure 6).^13^ GNAS gene, usually called as GNAS complex locus, has five main transcripts encoding proteins including the stimulatory G protein (Gsα), A/B transcript, XLαs, NESP55, and antisense GNAS transcript. These proteins act as important roles in several signaling pathways. As the most common product, the stimulatory G protein was extensively investigated participating in the process of transformation from ATP to cAMP and was primarily located in the plasma membrane.^14^ Based on the clinicopathology analysis and sequencing of oncogenes, mutations in GNAS along with Ras/Raf pathway tend to occur in invasive mucinous lung adenocarcinomas.^15^ This pro‐tumorigenic effect is postulated to converge with the effect of Cabozantinib target RET in ccRCC and GNAS gain may thus be associated with sensitivity to the drug^16^ (Figure 6).

Sensitivity to Pazopanib was associated with ACRBP gain and CTBP1 loss. Acrosin binding protein, encoded by ACRBP gene, is a kind of acrosomal matrix protein and takes part in decorating and packing acrosin zymogen. In humans, the ACRBP expression is usually limited in testes in general, however, it could be detected in various tumor types like bladder, lung, and breast cancer. From a molecular perspective, with ACRBP highly expressed, an abrogation of a mitotic spindle assembly was repressed and may elucidate the mechanism of sensitivity to paclitaxel. Moreover, high expression is related to poor survival and accelerated relapse.^17^ A postulated mechanistic link between ACRBP and Pazopanib target c‐Kit was that both of which play a role in mitotic spindle function, which exerts critical function in tumor cells^18^, ^19^ (Figure 6). CTBP1, together with CTBP2, was demonstrated as transcriptional corepressor that inhibits the expression of genes.^20^ The substrate‐binding domain (SBD) and the nucleotide‐binding domain (NBD) of CtBP1 are bind and each performs its own function.^21^ Sahu acknowledged that CtBP1 facilitates the epithelial‐mesenchymal transition (EMT) and then speed the progression of tumor development and metastasis.^22^ It was reported that CtBP1 could regulate the cell adhesion and connection, and NSG mice were companied with less metastasis when the CTBP1 gene deleted compared to control.^23^ We postulate that CTBP1 is associated with Pazopanib target PDGFR via ARF signaling^24^, ^25^ (Figure 6).

Sensitivity to Temsirolimus was associated with SULT1A3 gain and CDKN2A loss. SULT1A3 encodes the protein Sulfotransferase 1A3/1A4 to catalyze the sulfate amalgamation between phenolic agents and phenolic monoamines taking the 3'‐phospho‐5'‐adenylyl sulfate as sulfonate patron. A phenol sulfotransferase encoded by this gene is subject to destruction from heat. The Sulfotransferase 1A3 and 1A4 origins from the segmental duplication and differs from the splicing of transcripts. Although the metabolism of catecholamines and serotonin has been confirmed relevant to sulfoconjugation, a recent study focused on the single nucleotide polymorphisms of SULT1A3 gene and demonstrated the distinction of enzyme activities between them.^26^ Of note, SULT genes were established to participate in metabolism of a series of drugs. Whether their overexpression and copy number gain in tumor cells affects metabolism of compounds entering the cell remains unclear. Nonetheless, this tie theoretically explains in part sensitivity to mTORi ^27^(Figure 6). CDKN2A transcripts could be translated into two products, p16 and p14arf. The p16 was widely known as a tumor suppressor inhibiting the CDK4 and CDK6, impeding the process from G1 to S phase. Similarly, p14arf plays the same role in cell cycle by activating p53 tumor suppressor. Loss of CDKN2A function by mutation or copy number loss is a major landmark in a variety of cancers. An relatively mature regulatory pathway is therefore proposed for its sensitivity to mTORi, namely the MDM2/AMPK/TSC1 regulatory axis^28^ (Figure 6).

We showed CCNE1 gain was associated with Axitinib sensitivity. CCNE1 encodes the protein as the member of cyclin family and involved in the regulation of CDK activity. This cyclin mediates CDK2 and functions in the G1‐S phase, with the levels diminished following the cell cycle progression. It was reported that 14.8% of patients with ovarian clear cell carcinomas developed CCNE1 copy number gain, which in turns is correlated with poor overall survival and outcome.^29^ A postulated association between CCNE1 signaling and Axitinib target c‐Kit is via inhibition by MNK1/3^30^ (Figure 6). We also showed LRP10 loss was associated with Sunitinib sensitivity. LRP10 is involved in lipoprotein metabolism. LRP10 was also recognized as a prognostic marker for patients with hepatocellular carcinoma.^31^ It has been reported that LRP functions as a coreceptor that modulates signal transduction pathways initiated by the PDGFR (Figure 6).

Our study has limitations. First, the in silico findings should be validated in a variety of in vitro and in vivo studies to solidify the role of the candidate gene. However, as an exploratory study, we are now performing mechanistic analysis on select genes. Second, most of the candidate genes are theoretically remote to the drug targets and therefore functional analysis could be difficult.

## 5 CONCLUSION

Here we show CNVs of several genes that are associated with sensitivity and resistance to commonly used TKIs and mTORi in ccRCC, including ADCYAP1 loss and GNAS gain associated with sensitivity and resistance and to Cabozantinib, respectively; ACRBP gain and CTBP1 loss were associated with sensitivity and resistance and to Pazopanib, respectively; SULT1A3 gain and CDKN2A loss associated with resistance and sensitivity to the temsirolimus, respectively; CCNE1 gain associated with resistance to Axitinib, and LRP10 loss associated with resistance to Sunitinib.

## CONFLICT OF INTEREST

None.

## AUTHORS CONTRIBUTIONS

YL, YS, ZZ, HW, and CF performed experiments. CF and ZZ designed the study. YL and CF wrote the draft manuscript. ZZ and YS wrote the revised manuscript.

## Data Availability

Not applicable.
